# Supplementary material for: MAP1LC3C repression reduces CIITA- and HLA class II expression in non-small cell lung cancer
Source: PLoS One. 2025 Feb 10;20(2):e0316716. doi: 10.1371/journal.pone.0316716 (PMC11809862; doi:10.1371/journal.pone.0316716)
Supplement: S1 Fig — (PDF) [file pone.0316716.s001.pdf]

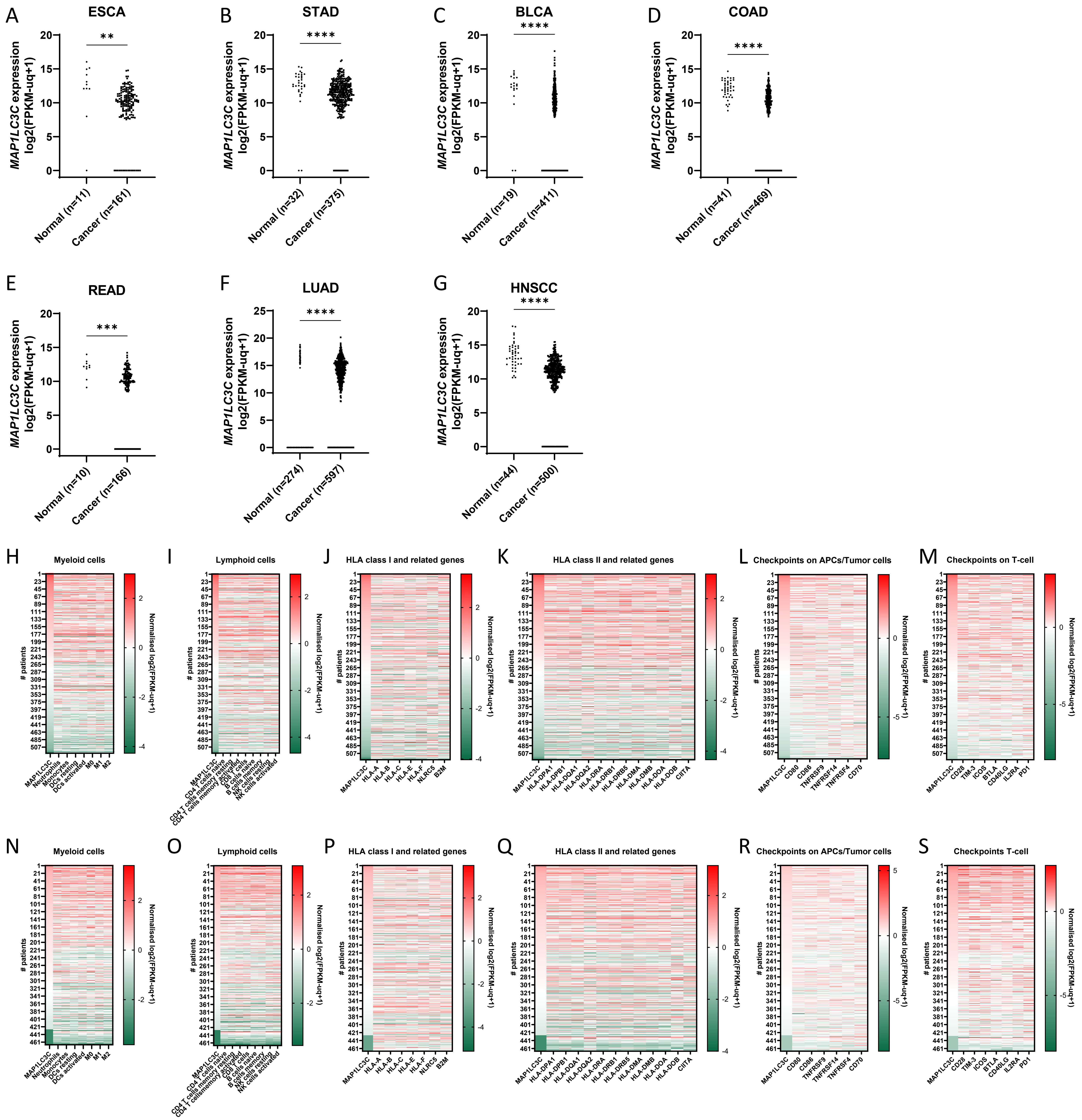

S1 Fig. *MAP1LC3C* expression decreases in several cancer types and correlates with low cancer immunity in LUAD and HNSCC, related to Figure 1. A-G) *MAP1LC3C* expression is decreased in cancer compared to normal tissue in the different TCGA cohorts (**p<0.01, ***p<0.001, ****p<0.0001). ESCA, esophageal carcinoma; STAD, stomach adenocarcinoma; BLCA, bladder carcinoma; COAD, colon adenocarcinoma; READ, rectum adenocarcinoma; LUAD, lung adenocarcinoma; HNSCC, head and neck squamous cell carcinoma. Data are represented as median. Heat maps of *MAP1LC3C* in LUAD, **H)** myeloid and **I)** lymphoid immune cells genes' signatures, **J)** HLA class I and **K)** HLA class II, HLA chaperones and CIITA, **L)** immune checkpoints on APCs/tumors and **M)** immune checkpoints on T-cells. Spearman correlation (r) values are indicated in Table 2. Heat maps of *MAP1LC3C* in HNSCC, **N)** myeloid and **O)** lymphoid immune cells genes' signatures, **P)** HLA class I and **Q)** HLA class II, HLA chaperones and CIITA, **R)** immune checkpoints on APCs/tumors and **S)** immune checkpoints on T-cells. FKPM, Fragments per Kilobase of transcript per million Mapped reads. Spearman correlation (r) values are indicated in Table 3.

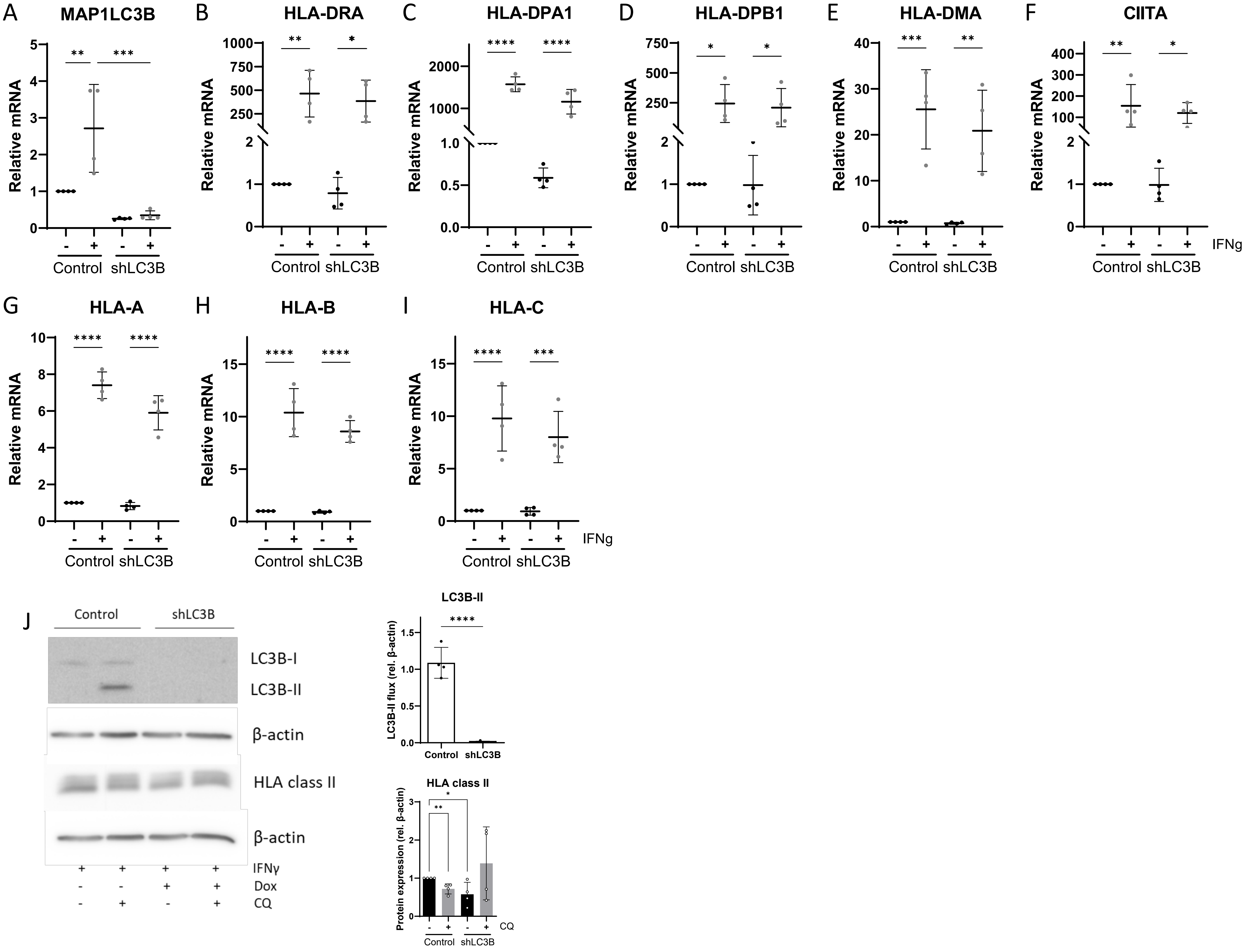

S2 Fig. Loss of MAP1LC3B does not affect HLA class I and class II expression, related to Figure 3. Quantitative PCR of **A)** *MAP1LC3B*, **B-D)** *HLA class II (HLA-DRA, HLA-DPA1, HLA-DPB1*), **E)** HLA chaperone (*HLA-DMA*), **F)** *CIITA* and **G-I)** *HLA class I (HLA-A, HLA-B and HLA-C)* expression following IFNγ (*p<0.05, **p<0.01, ***p<0.001, ****p<0.0001). **J)** Immunoblot analysis and quantification of MAP1LC3B and HLA class II protein following IFNγ and/or chloroquine (CQ) and/or doxycycline (dox) treatment (*p<0.05, **p<0.01, ****p<0.0001). Data are representative of three independent experiments and values are expressed as mean ± SD.

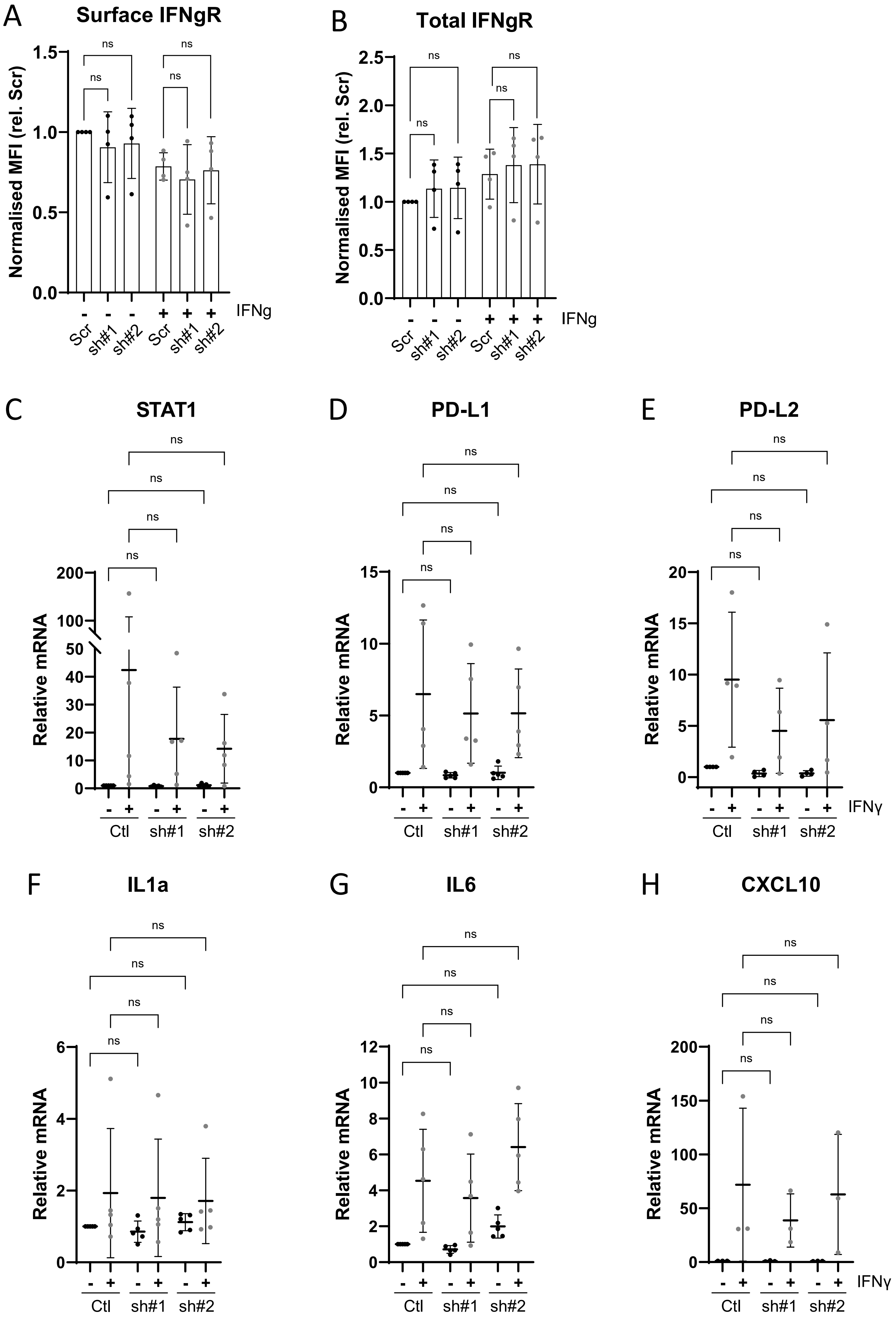

S3 Fig. Loss of MAP1LC3C does not affect IFNγ pathway, related to Figure 3. Flow cytometric analysis of IFNγ receptor (IFNγR) protein expression at **A)** the plasma membrane (surface) and **B)** total expression after cell permeabilization (total) following IFNγ treatment. Quantitative PCR of **C-H)** Interferon-Stimulated Gene (ISG) expression following IFNγ treatment (ns: non-significant, **p<0.01). Data are representative of three or more independent experiments and values are expressed as mean ± SD.

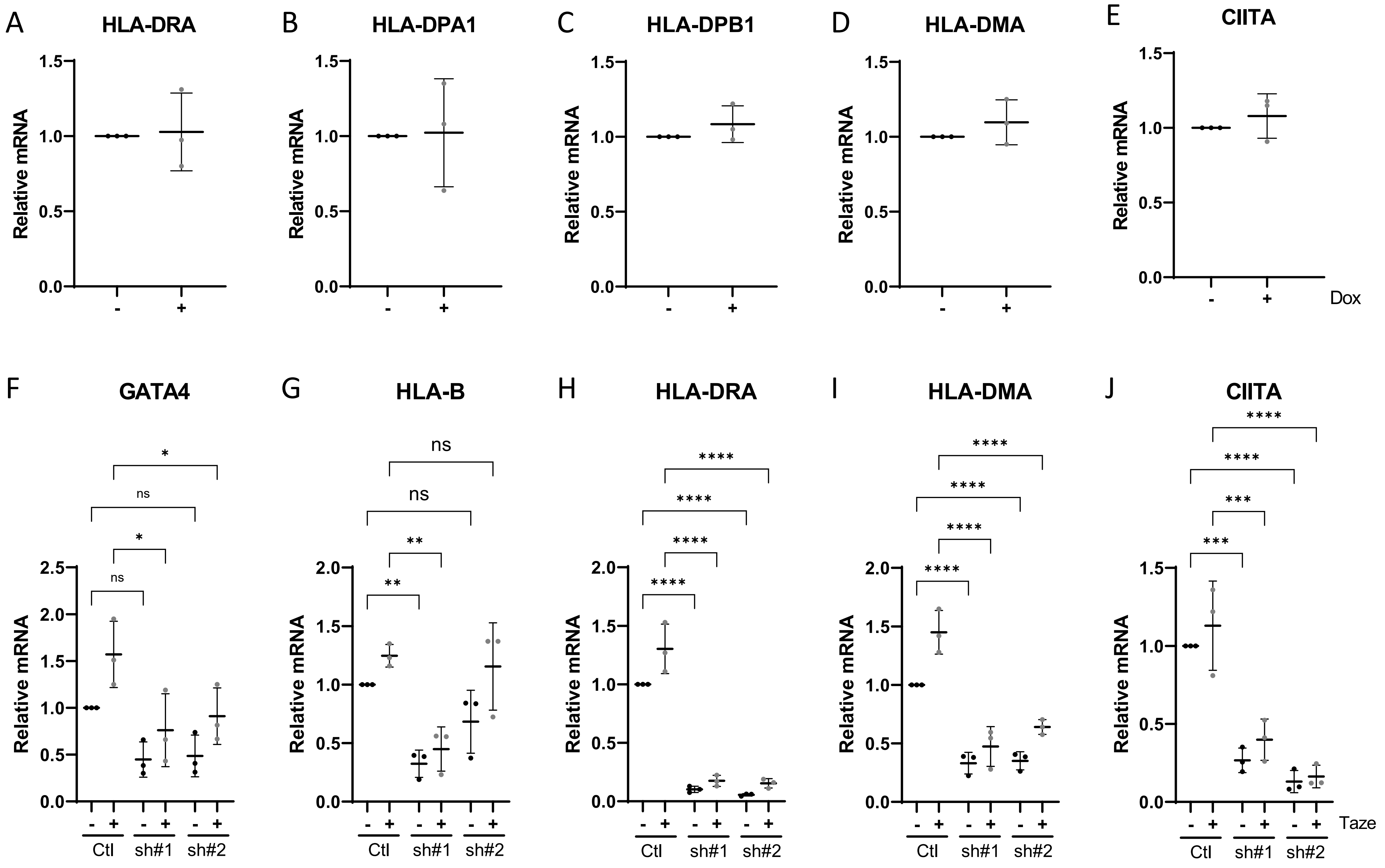

S4 Fig. Doxycycline and PRC2 complex are not involved in *HLA class I and II* expression impairment in MAP1LC3C deficient cells, related to Figure 4. Quantitative PCR of A-C) *HLA class II* (*HLA-DRA, HLA-DPA1, HLA-DPB1*), D) HLA chaperone (*HLA-DMA*) and E) *CIITA* expression following doxycycline treatment in control cells. Quantitative PCR of F-G) PRC2 target genes (*GATA4* and *HLA-B*), H) *HLA class II* (*HLA-DRA*), I) HLA chaperone (*HLA-DMA*) and J) *CIITA* expression following PRC2 inhibition (ns: non-significant, *p<0.05, **p<0.01, ***p<0.001, ****p<0.0001). Data are representative of three independent experiments and values are expressed as mean ± SD.

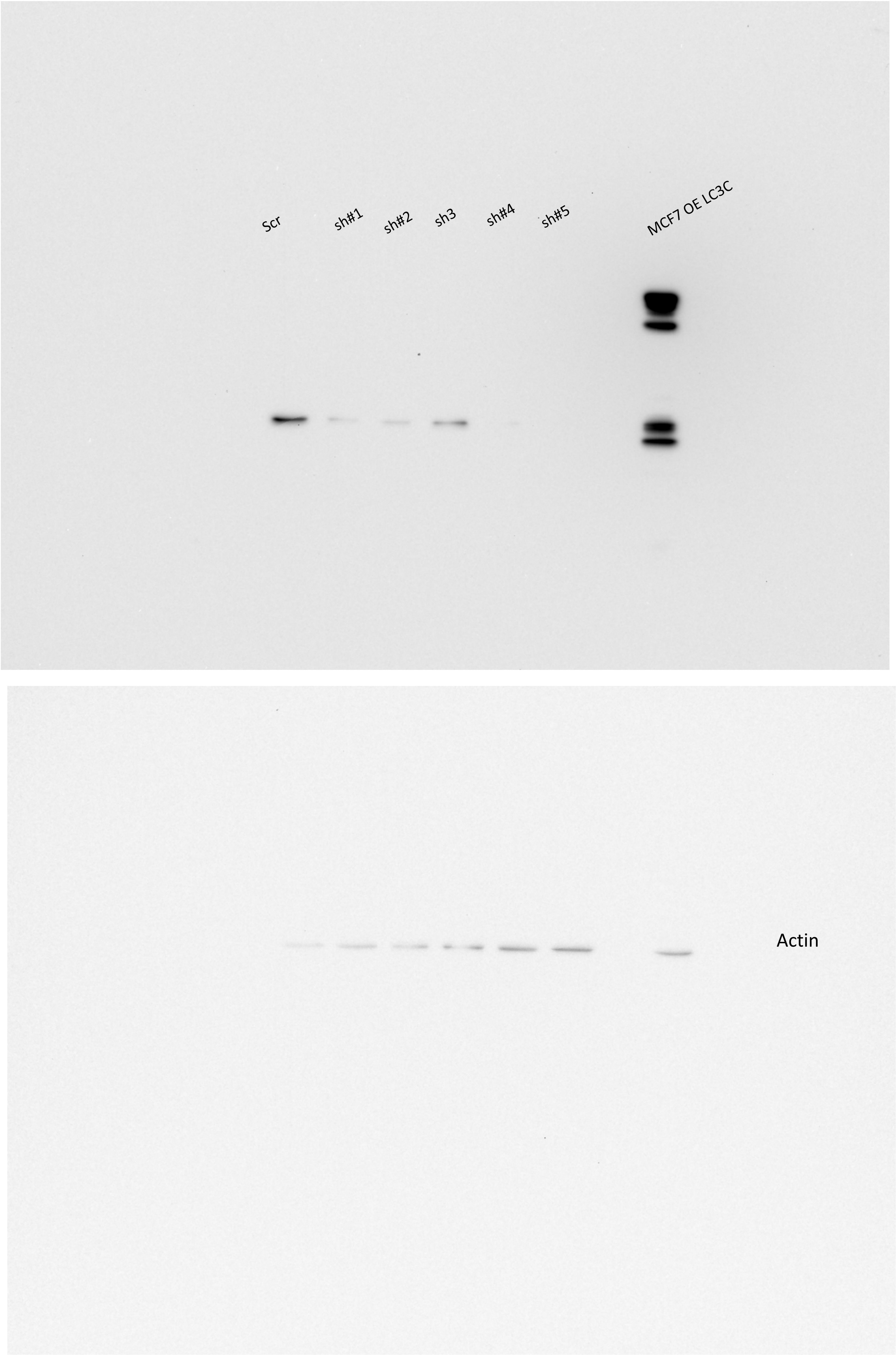

S5,1 Fig. Uncropped and unadjusted blots (light sensitive films) Fig 3A. (Top) LC3C antibody on 5 independent shRNA. For identification MCF7 cells with ectopic LC3C Overexpression were loaded.

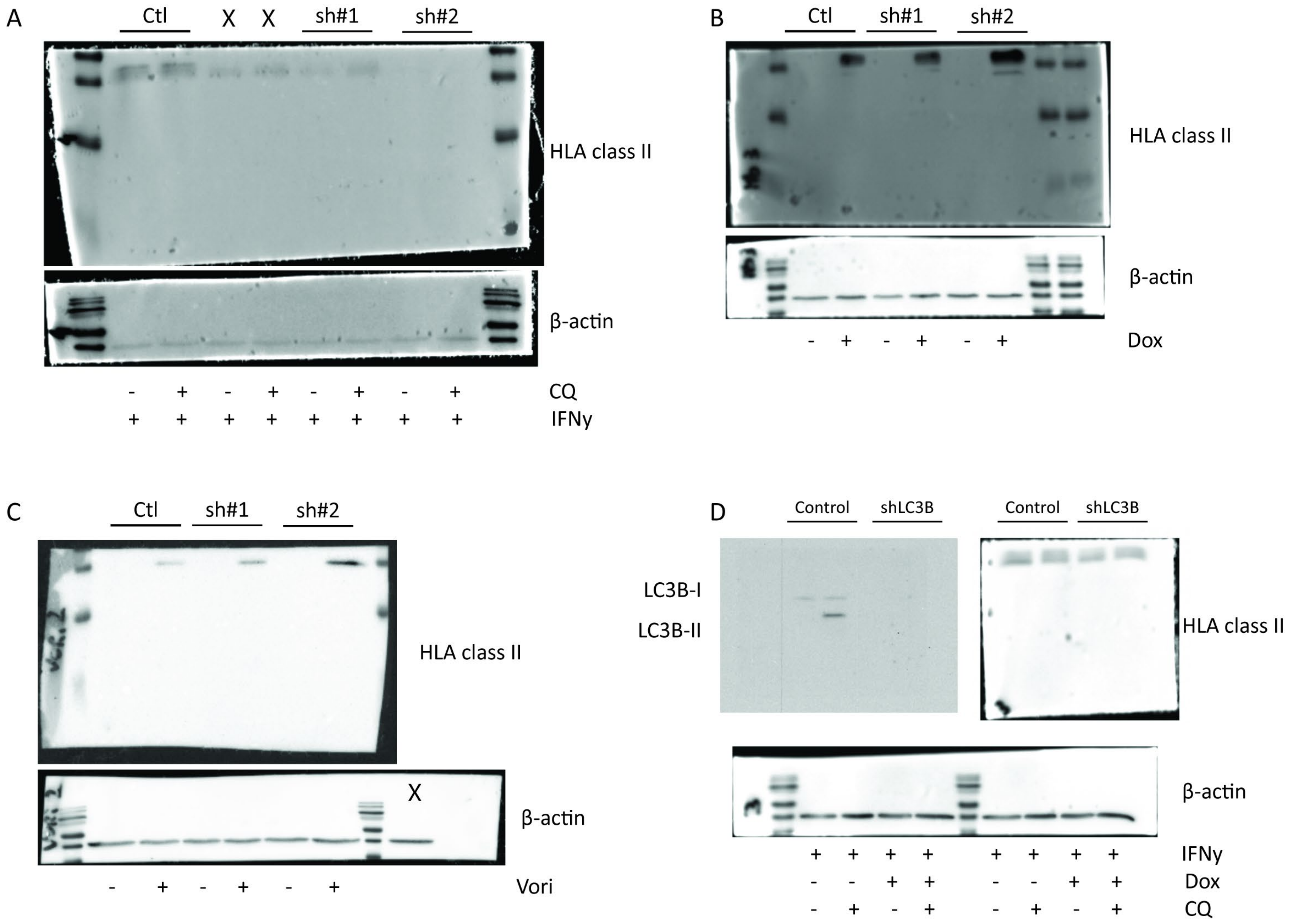

S5,2 Fig. Uncropped and unadjusted blots (digital image acquisition). A) Uncropped blot Fig 3K. B) Uncropped blot Fig 4G. C) Uncropped blot Fig 4M. D) Uncropped blot S2J Fig.
