# Supplementary material for: MAP1LC3C repression reduces CIITA- and HLA class II expression in non-small cell lung cancer
Source: PLoS One. 2025 Feb 10;20(2):e0316716. doi: 10.1371/journal.pone.0316716 (PMC11809862; doi:10.1371/journal.pone.0316716)
Supplement: S2 Fig — (PDF) [file pone.0316716.s002.pdf]

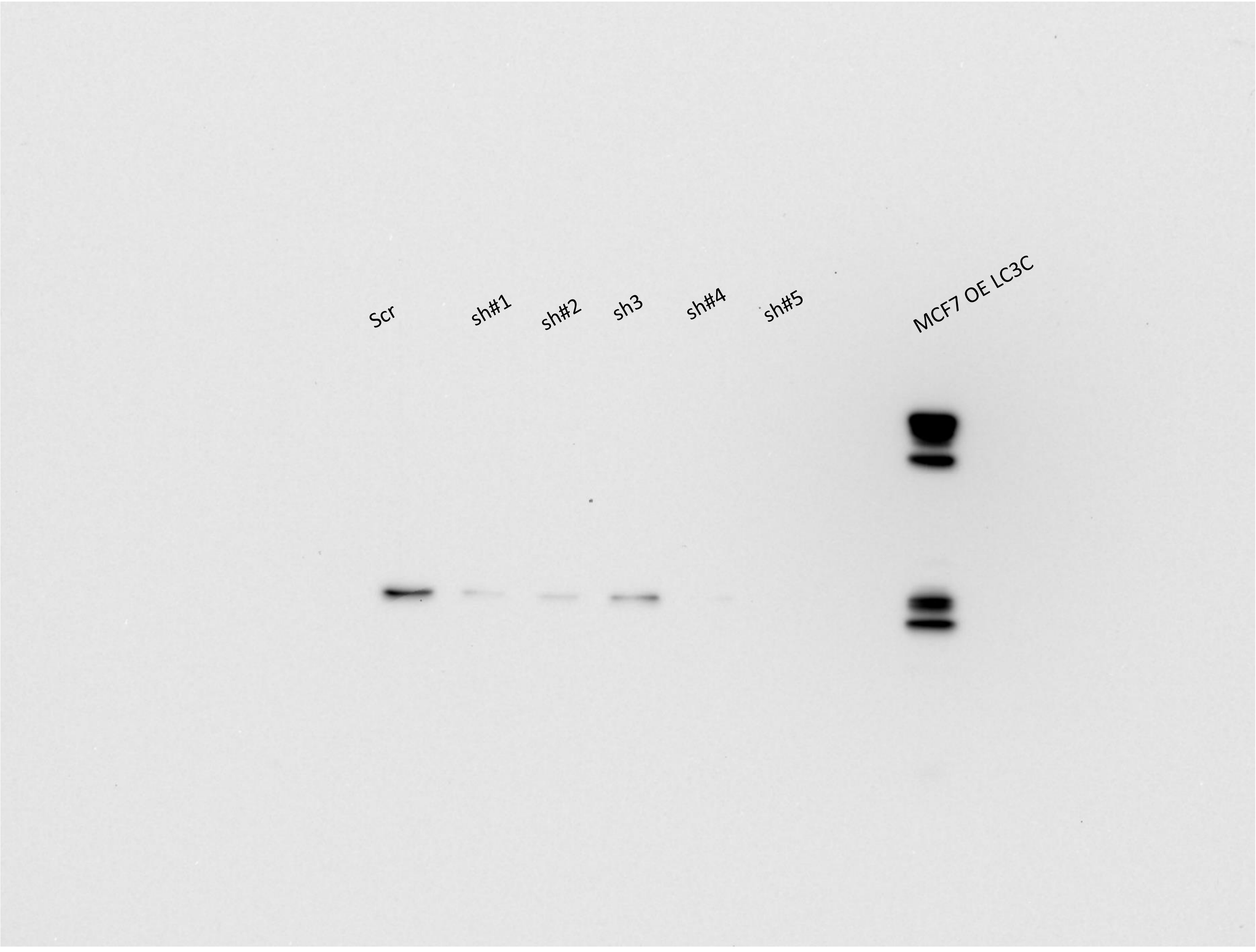

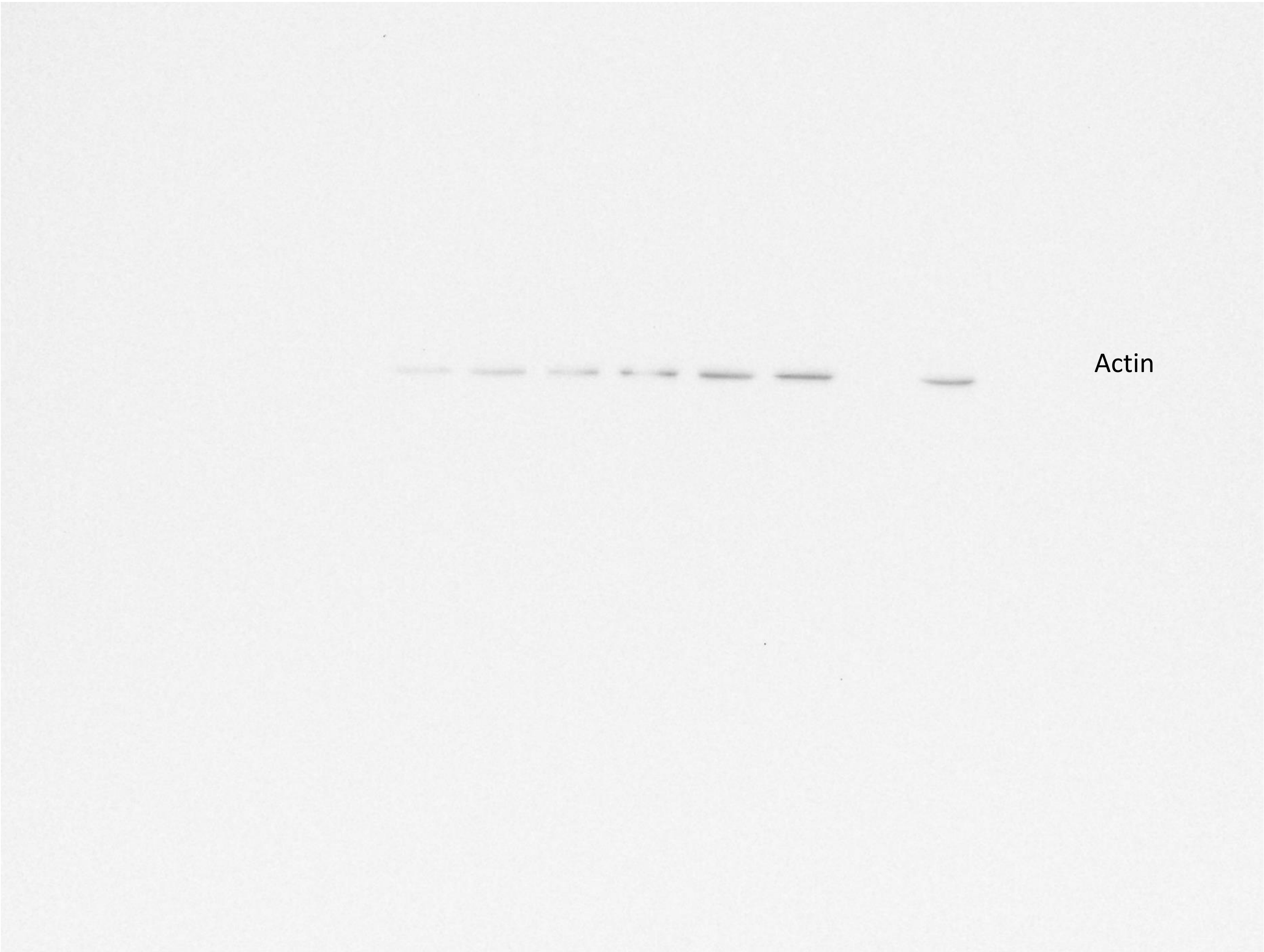

S1,1 Fig. Uncropped and unadjusted blots (light sensitive films) Fig 3A. (Top) LC3C antibody on 5 independent shRNA. For identification MCF7 cells with ectopic LC3C Overexpression were loaded.

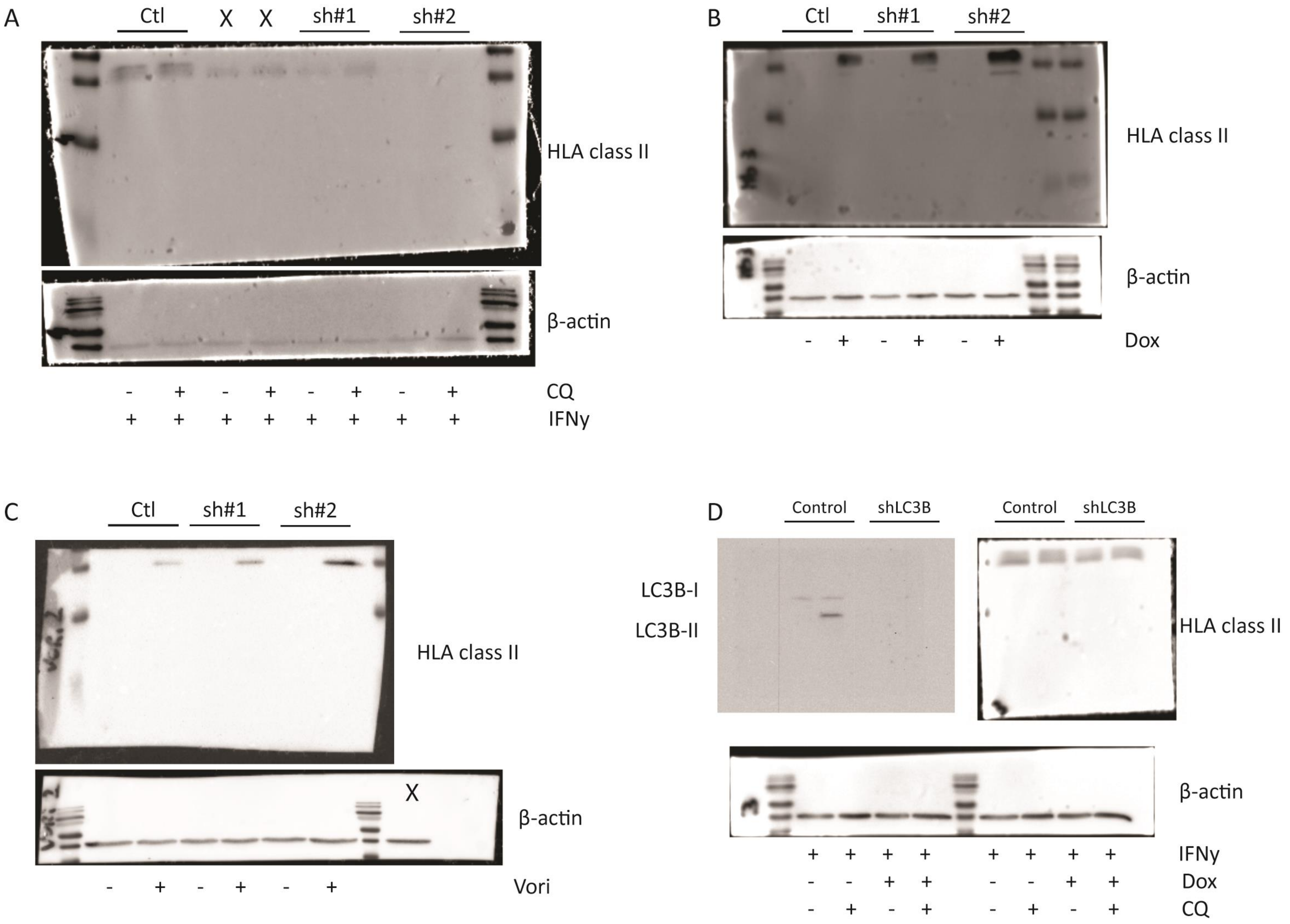

S1,2 Fig. Uncropped and unadjusted blots (digital image acquisition). A) Uncropped blot Fig 3K. B) Uncropped blot Fig 4G. C) Uncropped blot Fig 4M. D) Uncropped blot S2J Fig.
