# Supplementary material for: MAP1LC3C repression reduces CIITA- and HLA class II expression in non-small cell lung cancer
Source: PLoS One. 2025 Feb 10;20(2):e0316716. doi: 10.1371/journal.pone.0316716 (PMC11809862; doi:10.1371/journal.pone.0316716)
Supplement: S1 Table — (DOCX) [file pone.0316716.s003.docx]

**SUPPLEMENTAL FIGURES**

**Table S1. Spearman correlation (r) values between *MAP1LC3C* and cancer immunity genes expression are indicated in LUAD, related to Figure 1.** *: 0.5 < r < 0.6.

| Myeloid cells | Neutrophils | Monocytes | DCs resting | DCs activated | M0 | M1 | M2 |  |
| --- | --- | --- | --- | --- | --- | --- | --- | --- |
| Spearman r | 0.3754 | 0.4874 | 0.5442* | 0.4746 | 0.3943 | 0.3433 | 0.4905 |  |
|  |  |  |  |  |  |  |  |  |
| Lymphoid cells | **CD4 T cells naïve** | **CD4 T cells memory resting** | **CD4 T cells memory activated** | **CD8 T cells** | **B cells naïve** | **B cells memory** | **NK cells resting** | **NK cells activated** |
| Spearman r | 0.3663 | 0.3391 | 0.2625 | 0.2920 | 0.2957 | 0.3010 | 0.3302 | 0.2764 |
|  |  |  |  |  |  |  |  |  |
| HLA class I and related genes | **HLA-A** | **HLA-B** | **HLA-C** | **HLA-E** | **HLA-F** | **NLRC5** | **B2M** |  |
| Spearman r | 0.1595 | 0.2535 | 0.2279 | 0.2862 | 0.2268 | 0.1582 | 0.2876 |  |
|  |  |  |  |  |  |  |  |  |
| HLA class II and related genes | **HLA-DPA1** | **HLA-DPB1** | **HLA-DQA1** | **HLA-DQA2** | **HLA-DRA** | **HLA-DRB1** | **HLA-DRB5** |  |
| Spearman r | 0.4638 | 0.4343 | 0.3996 | 0.3175 | 0.4771 | 0.4142 | 0.448 |  |
|  | **HLA-DMA** | **HLA-DMB** | **HLA-DOA** | **HLA-DOB** | **CIITA** |  |  |  |
|  | 0.3833 | 0.4611 | 0.4109 | 0.3211 | 0.3369 |  |  |  |
|  |  |  |  |  |  |  |  |  |
| Checkpoints on APCs and tumor cells | **CD80** | **CD86** | **TNFRSF9** | **TNFRSF14** | **TNFRSF4** | **CD70** |  |  |
| Spearman r | 0.4676 | 0.3922 | 0.2990 | 0.1968 | 0.1818 | 0.2252 |  |  |
|  |  |  |  |  |  |  |  |  |
| Checkpoints on T-cell | **CD28** | **TIM-3** | **ICOS** | **BTLA** | **CD40LG** | **IL2RA** | **PD1** |  |
| Spearman r | 0.3969 | 0.4125 | 0.3666 | 0.2963 | 0.3384 | 0.274 | 0.2112 |  |

**Table S2. Spearman correlation (r) values between *MAP1LC3C* and cancer immunity genes expression are indicated in HNSCC, related to Figure 1.** *: 0.5 < r < 0.6; **: r > 0.6.

| Myeloid cells | Neutrophils | Monocytes | DCs resting | DCs activated | M0 | M1 | M2 |  |
| --- | --- | --- | --- | --- | --- | --- | --- | --- |
| Spearman r | 0.4817 | 0.5476* | 0.5891* | 0.4963 | 0.4829 | 0.4344 | 0.6039** |  |
|  |  |  |  |  |  |  |  |  |
| Lymphoid cells | **CD4 T cells naïve** | **CD4 T cells memory resting** | **CD4 T cells memory activated** | **CD8 T cells** | **B cells naïve** | **B cells memory** | **NK cells resting** | **NK cells activated** |
| Spearman r | 0.5349* | 0.5172* | 0.4318 | 0.4722 | 0.5419* | 0.5295* | 0.4672 | 0.4251 |
|  |  |  |  |  |  |  |  |  |
| HLA class I and related genes | **HLA-A** | **HLA-B** | **HLA-C** | **HLA-E** | **HLA-F** | **NLRC5** | **B2M** |  |
| Spearman r | 0.01032 | 0.0832 | 0.1033 | 0.1114 | 0.01948 | 0.1151 | 0.1086 |  |
|  |  |  |  |  |  |  |  |  |
| HLA class II and related genes | **HLA-DPA1** | **HLA-DPB1** | **HLA-DQA1** | **HLA-DQA2** | **HLA-DRA** | **HLA-DRB1** | **HLA-DRB5** |  |
| Spearman r | 0.4602 | 0.3642 | 0.4874 | 0.3036 | 0.4577 | 0.4016 | 0.3625 |  |
|  | **HLA-DMA** | **HLA-DMB** | **HLA-DOA** | **HLA-DOB** | **CIITA** |  |  |  |
|  | 0.396 | 0.4588 | 0.4966 | 0.2572 | 0.4119 |  |  |  |
|  |  |  |  |  |  |  |  |  |
| Checkpoint on APCs and tumor cells | **CD80** | **CD86** | **TNFRSF9** | **TNFRSF14** | **TNFRSF4** | **CD70** |  |  |
| Spearman r | 0.3647 | 0.3703 | 0.4341 | 0.2071 | 0.3109 | 0.1198 |  |  |
|  |  |  |  |  |  |  |  |  |
| Checkpoints T-cell | **CD28** | **TIM-3** | **ICOS** | **BTLA** | **CD40LG** | **IL2RA** | **PD1** |  |
| Spearman r | 0.5967* | 0.4241 | 0.4106 | 0.5167* | 0.5201* | 0.5045* | 0.3506 |  |

**Table S3. Differentially expressed genes (DEGs) used to define myeloid and lymphoid progenitor cells, related to START Methods.**

| Cell types | Genes name |
| --- | --- |
| NK cells resting | AZU1; BPI; CAMP; CCL5; CD160; CD2; CD244; CD247; CD7; CD96; CDHR1; CEACAM8; CST7; CTSW; DEFA4; ELANE; GFI1; GNLY; GZMA; GZMB; GZMH; GZMK; GZMM; IL12RB2; IL18R1; IL18RAP; IL2RB; KIR2DL1; KIR3DL2; KLRB1; KLRC3; KLRC4; KLRD1; KLRF1; KLRK1; LCK; MGAM; MS4A3; NAALADL1; NKG7; NME8; PLEKHF1; PRF1; PRR5L; PTGDR; PTPRCAP; PVRIG; S1PR5; SH2D1A; TBX21; TEP1; TRBC1; TRDC; TTC38; TXK; ZAP70; ZNF135 |
| NK cells activated | APOBEC3G; APOL6; CCL4; CCL5; CCND2; CD244; CD247; CD69; CD7; CD96; CDK6; CSF2; CST7; CTSW; DPP4; FASLG; GNLY; GPR171; GPR18; GRAP2; GZMA; GZMB; GZMH; GZMM; IFNG; IL12RB2; IL18R1; IL18RAP; IL2RB; KIR2DL1; KIR2DL4; KIR2DS4; KIR3DL2; KLRB1; KLRC3; KLRD1; KLRF1; KLRK1; LCK; LTA; LTB; NAALADL1; NCR3; NKG7; OSM; PRF1; PRR5L; PTGDR; PTGER2; PTPRCAP; PVRIG; S1PR5; SH2D1A; SOCS1; TBX21; TNFSF14; TRDC; TXK; ZAP70 |
| B cells naïve | ABCB4; ADAM28; BACH2; BANK1; BCL7A; BEND5; BLK; BRAF; CD180; CD19; CD1C; CD22; CD37; CD69; CD72; CD79A; CD79B; CR2; CXCR5; EAF2; FAIM3; FCER2; FCGR2B; FCRL2; FRK; GPR18; GUSBP11; HHEX; HLA-DOB; IGHD; IGHM; IGKC; IGLL3P; IL4R; IRF8; KIAA0226L; LINC00921; LTB; LY86; MEP1A; MICAL3; MS4A1; NIPSNAP3B; NMBR; P2RX5; P2RY14; PNOC; PSG2; PTPRCAP; RALGPS2; RASGRP2; SELL; SIK1; SLC12A1; SPIB; STAP1; TCL1A; UGT1A8; VPREB3; ZNF286A |
| B cells memory | ADAM28; AIM2; ALOX5; BACH2; BANK1; BLK; CCR6; CD180; CD19; CD1C; CD22; CD27; CD37; CD69; CD72; CD79A; CD79B; CLCA3P; CR2; CXCR5; DENND5B; FAIM3; FAM65B; FCGR2B; FCRL2; FRK; GNG7; GPR18; GUSBP11; HHEX; HLA-DOB; IFNA10; IGHD; IGHM; IGKC; IGLL3P; IL7; IRF8; KIAA0226L; LTB; LY86; MBL2; MS4A1; NMBR; NPIPB15; P2RX5; PNOC; PTPRCAP; RALGPS2; RASGRP2; SIK1; SIT1; SLC12A1; SP140; SPIB; STAP1; TMEM156; TNFRSF13B; TNFRSF17; TRAF4; VPREB3; ZBTB32 |
| CD8 T cells | BCL11B; CCL5; CD2; CD247; CD27; CD3D; CD3E; CD3G; CD6; CD69; CD7; CD8A; CD8B; CD96; CRTAM; CST7; CTSW; DPP4; DSC1; DUSP2; FAIM3; FLT3LG; GNLY; GPR171; GRAP2; GZMA; GZMB; GZMH; GZMK; GZMM; ICOS; IGKC; IL7R; ITK; KLRB1; KLRC3; KLRC4; KLRD1; KLRF1; KLRK1; LAG3; LCK; LEF1; LIME1; LTB; LY9; MAP4K1; MAP9; NCR3; NKG7; PIK3IP1; PRF1; PTGDR; PTPRCAP; PVRIG; RASA3; RPL3P7; SH2D1A; SIRPG; TCF7; TRAC; TRAT1; TRAV12-2; TRAV13-1; TRBC1; TRDC; UBASH3A; ZAP70 |
| CD4 T cells naïve | ACAP1; ANKRD55; ATHL1; BCL11B; CCR7; CD2; CD247; CD27; CD3D; CD3G; CD40LG; CD7; CXorf57; DPP4; DSC1; EPHA1; FAIM3; FLJ13197; FLT3LG; GAL3ST4; GALR1; GPR1; GRAP2; GZMM; ICOS; IL7R; ITK; LAT; LCK; LEF1; LIME1; LTB; LY9; MAP4K1; MAP4K2; MAP9; RASGRP2; RPL3P7; SERGEF; SH2D1A; SIRPG; TCF7; TRAC; TRAT1; TRAV13-1; TRBC1; UBASH3A; VILL; WNT7A; ZAP70; ZNF204P; ZNF324 |
| CD4 T cells memory resting | BCL11B; CCL5; CCR6; CD2; CD247; CD27; CD28; CD3D; CD3E; CD3G; CD4; CD40LG; CD6; CD69; CD7; CD96; CTLA4; CTSW; DGKA; DPP4; EPB41; ETS1; FAIM3; FBXL8; FLT3LG; GPR171; GPR25; GRAP2; GZMA; GZMK; GZMM; ICOS; IL7R; ITK; KLRB1; LCK; LEF1; LIME1; LTB; LY9; NKG7; PBXIP1; PTGER2; PTPRCAP; RASA3; RASGRP2; RCAN3; RPL10L; RPL3P7; SH2D1A; SIRPG; ST8SIA1; TCF7; TRAC; TRAT1; TRAV13-1; TRAV13-2; TRAV21; TRAV8-6; TRAV9-2; TRBC1; UBASH3A; ZAP70; ZFP36L2 |
| CD4 T cells memory activated | CCL20; CD2; CD247; CD28; CD3D; CD3G; CD40LG; CD6; CD7; CDC25A; CSF2; CTLA4; CXCL13; DPP4; GPR171; GPR19; GZMB; ICOS; IFNG; IL12RB2; IL17A; IL26; IL2RA; IL3; IL4; IL9; LAG3; LCK; LTA; NKG7; ORC1; PMCH; RRP9; SH2D1A; SKA1; TNFRSF4; TNIP3; TRAC; TRAT1; UBASH3A |
| Monocytes | AIF1; APOBEC3A; AQP9; ASGR1; ASGR2; BST1; C5AR1; CCR2; CD1D; CD33; CD68; CDA; CFP; CHST15; CLEC4A; CLEC7A; CREB5; CSF3R; FAM198B; FCN1; FES; FOSB; FPR1; FZD2; HCK; HK3; HNMT; HPSE; IGSF6; LILRA2; LILRA3; LILRB2; LST1; MEFV; MNDA; MS4A6A; NCF2; NFE2; NLRP3; NOD2; P2RY13; PADI4; RNASE2; RNASE6; S100A12; SLC15A3; TLR2; TLR7; TLR8; UPK3A; VNN1; VNN2 |
| Macrophages M0 | ACP5; ADAMDEC1; AQP9; BHLHE41; C5AR1; CCDC102B; CCL18; CCL22; CCL7; CD68; CHI3L1; COL8A2; CSF1; CXCL3; CXCL5; CYP27A1; DCSTAMP; FAM198B; GPC4; HK3; IGSF6; MARCO; MMP9; NCF2; PLA2G7; PPBP; QPCT; SLAMF8; SLC12A8; SLC15A3; TNFSF14; TREM2; VNN1 |
| Macrophages M1 | ACHE; ADAMDEC1; APOBEC3A; APOL3; APOL6; AQP9; ARRB1; CCL19; CCL5; CCL8; CCR7; CD38; CD40; CHI3L1; CLIC2; CXCL10; CXCL11; CXCL13; CXCL9; CYP27B1; DHX58; EBI3; GGT5; HESX1; IDO1; IFI44L; IL2RA; KIAA0754; KYNU; LAG3; LAMP3; LILRA3; LILRB2; NOD2; PLA1A; PTGIR; RASSF4; RSAD2; SIGLEC1; SLAMF1; SLC15A3; SLC2A6; SOCS1; TLR7; TLR8; TNFAIP6; TNIP3; TRPM4 |
| Macrophages M2 | ADAMDEC1; AIF1; ALOX15; CCL13; CCL14; CCL18; CCL23; CCL8; CD209; CD4; CD68; CFP; CHI3L1; CLEC10A; CLEC4A; CLIC2; CRYBB1; EBI3; FAM198B; FES; FRMD4A; FZD2; GGT5; GSTT1; HRH1; HTR2B; MS4A6A; NME8; NPL; P2RY13; PDCD1LG2; RENBP; SIGLEC1; SLC15A3; TLR8; TREM2; WNT5B |
| DCs resting | ACP5; AIF1; ALOX15; C1orf54; CCDC102B; CCL13; CCL17; CCL18; CCL22; CD1A; CD1B; CD1C; CD1E; CD209; CD33; CD68; CLEC10A; CLEC4A; CLEC7A; CLIC2; DHRS11; EGR2; FAM198B; FCER1A; FCER2; FLVCR2; FPR3; FZD2; HLA-DQA1; IGSF6; MMP12; NCF2; PLA2G7; PPFIBP1; RNASE6; SCN9A; SLAMF8; SLC15A3; TMEM255A; TREM2 |
| DCs activated | ARHGAP22; BIRC3; CCL1; CCL13; CCL17; CCL18; CCL19; CCL20; CCL22; CCL5; CCL8; CCR7; CD1B; CD1E; CD80; CD86; CHST7; CLIC2; CXCL10; CXCL11; CYP27A1; DHX58; EBI3; ETV3; FPR3; HESX1; HTR2B; IDO1; IFI44L; IL12B; IL2RA; KYNU; LAMP3; MAP3K13; MMP12; MSC; NR4A3; PDCD1LG2; PLA1A; PLA2G7; PTGIR; RASSF4; RSAD2; SIGLEC1; SLC15A3; SLC2A6; SLCO5A1; ST3GAL6; TMEM255A; TNFAIP6; TNFRSF11A; TNFRSF4; TREM2 |
| Neutrophils | AIF1; APOBEC3A; AQP9; BTNL8; C5AR1; CAMP; CASP5; CCR3; CDA; CEACAM3; CFP; CHI3L1; CHST15; CLC; CREB5; CSF3R; CXCR1; CXCR2; DPEP2; EMR2; EMR3; FAM212B; FCGR3B; FFAR2; FPR1; FPR2; GPR97; HAL; HSPA6; IGSF6; IL18RAP; LILRA2; LILRB2; LST1; MAK; MEFV; MGAM; MMP25; MNDA; MXD1; NCF2; NFE2; P2RY13; P2RY14; PADI4; PGLYRP1; PLEKHG3; QPCT; REPS2; S100A12; STEAP4; TLR2; TLR8; TNFAIP6; TNFRSF10C; TREM1; TREML2; VNN1; VNN2; VNN3 |

**Table S4. Quantitative real-time PCR primer sequences, related to STAR Methods.**

| Target genes | Forward (5’ – 3’) | Reverse (5’ – 3’) |
| --- | --- | --- |
| *RPL13A* | CCGGGTTGGCTGGAAAGGTAATTATG | CTTCTCGGCCTGTTTCCGTAC |
| *HLA-DPA1* | GAGCTGTGATCTTGAGAGC | CTGTTGGTCTATGCGTCTGTAC |
| *HLA-DMA* | GTGTGGCAAGAAGGTATGGG | GTCATCTGGCCACATTGGAGT |
| *HLA-DRA* | GCCATAAGTGGAGTCCCTGT | CGCCTGATTGGTCAGGATTC |
| *HLA-DPB1* | GCTCTGACGGCGTTACTGAT | GCGCTGTGTCCCATTAAACG |
| *HLA-A* | ACAGACTGACCGAGTGGA | CACGTCGCAGCCATACATTATC |
| *HLA-B* | TAGCAGTTGTGGTCATCGGA | ACAGCTGTCTCAGGCTTTTCAA |
| *HLA-C* | ACACAGAAGTACAAGCGCCA | CGTAGGCGGACTGGTCATAC |
| *CIITA* | CCTGGAGCTTCTTAACAGCGA | TGTGTCGGGTTCTGAGTAGAG |
| *PD-L1* | GGCATTTGCTGAACGCATTT | GGTCTTCCTCTCCATGCACAA |
| *PD-L2* | ACCAGTGTTCTGCGCCTAAA | CCTGGGTTCCATCTGACTTTGA |
| *CXCL10* | TGATGGCCTTCGATTCTGGATT | GTGGCATTCAAGGAGTACCTC |
| *IL1A* | AGATGCCTGAGATACCCAAAACC | CCAAGCACACCCAGTAGTCT |
| *IL6* | CCTGAACCTTCCAAAGATGGC | TTCACCAGGCAAGTCTCCTCA |
| *STAT1* | GGCAAAGAGTGATCAGAAACAA | GTTCAGTGACATTCAGCAACTC |
| *MAP1LC3B* | AACGGGCTGTGTGAGAAAAC | AGTGAGGACTTTGGGTGTGG |
| *MAP1LC3C* | AAACCAAGTTCCTGGTCCCG | ACACGAAGCCATCCTCATCC |
| *GATA4* | GTGTCCCAGACGTTCTCAGTC | GGGAGACGCATAGCCTTGT |
| *c-Myc* | GGTCTTTTCATTGTTTTCCA | TCAAGAGGCGAACACACAAC |

**Table S5. TaqMan probes, related to STAR Methods.**

| Target genes | Assay ID TaqMan |
| --- | --- |
| *18S* | Hs03928990_g1 |
| *MAP1LC3C* | Hs01374916_m1 |
